# Poor post‐exposure prophylaxis completion despite improvements in post‐violence service delivery in 14 PEPFAR‐supported sub‐Saharan African countries, 2018–2023

**DOI:** 10.1002/jia2.26469

**Published:** 2025-06-26

**Authors:** Udhayashankar Kanagasabai, Stephanie M. Davis, Viva Thorsen, Emily Rowlinson, Anne Laterra, Jennifer Hegle, Carrine Angumua, Alexandre Ekra, Minlangu Mpingulu, Meklit Getahun, Fikirte Sida, Phumzile Mndzebele, Caroline Kambona, Puleng Ramphalla, Eunice Mtingwi, Wezi Msungama, Meghan Duffy, Bukola Adewumi, Ezeomu Olotu, Jackson Sebeza, Jane Kitalile, Rose Apondi, Carlos Muleya, Meagan Cain

**Affiliations:** ^1^ HIV Prevention Branch, Division of Global HIV and Tuberculosis, CDC Atlanta Georgia USA; ^2^ Division of Global HIV and Tuberculosis, CDC Yaounde Cameroon; ^3^ Division of Global HIV and Tuberculosis, CDC Abidjan Cote d'Ivoire; ^4^ Division of Global HIV and Tuberculosis, CDC Kinshasa Democratic Republic of the Congo; ^5^ Division of Global HIV and Tuberculosis, CDC Addis Ababa Ethiopia; ^6^ Division of Global HIV and Tuberculosis, CDC Mbabane Eswatini; ^7^ Division of Global HIV and Tuberculosis, CDC Nairobi Kenya; ^8^ Division of Global HIV and Tuberculosis, CDC Maseru Lesotho; ^9^ Division of Global HIV and Tuberculosis, CDC Lilongwe Malawi; ^10^ Division of Global HIV and Tuberculosis, CDC Maputo Mozambique; ^11^ Division of Global HIV and Tuberculosis, CDC Abuja Nigeria; ^12^ Division of Global HIV and Tuberculosis, CDC Kigali Rwanda; ^13^ Division of Global HIV and Tuberculosis, CDC Dar es Salam Tanzania; ^14^ Division of Global HIV and Tuberculosis, CDC Kampala Uganda; ^15^ Division of Global HIV and Tuberculosis, CDC Lusaka Zambia

**Keywords:** Africa, HIV prevention, HIV, intimate partner violence, post‐exposure prophylaxis, sexual violence

## Abstract

**Introduction:**

Sexual violence (SV) affects millions globally and has a well‐documented bidirectional association with HIV. Post‐exposure prophylaxis (PEP) is a critical, yet often underutilized, HIV prevention tool in post‐SV care. Despite its potential impact to reduce HIV transmission, SV care remains an overlooked service delivery point for HIV prevention. The U.S. Centers for Disease Control and Prevention (CDC), as part of the President's Emergency Plan for AIDS Relief (PEPFAR), supports PEP provision within broader post‐violence care (PVC) services. Understanding PEP utilization is crucial for optimizing service delivery and HIV prevention efforts.

**Methods:**

Using Monitoring Evaluation and Reporting data from fiscal years 2018–2023, we conducted a descriptive analysis of clients who received PVC and SV services through CDC‐supported programming in 14 sub‐Saharan African countries.

**Results:**

From 2018 to 2023, the annual number of clients receiving any PVC, and specifically SV, services increased by 233% (in 2018, *n* = 206,764; in 2023, *n* = 689,349) and 163% (in 2018, *n* = 42,848; in 2023, *n* = 112,838), respectively. Fewer than half of SV clients completed PEP (38% in 2018, *n* = 16,103; 31% in 2023, *n* = 35,118). Across all years combined, most SV clients (female: 185,414; male: 59,618) were aged 15–19 years. The age band and sex with the lowest proportion of clients completing PEP were males aged 15–19 (4%, *n* = 2296).

**Conclusions:**

The findings underscore a critical gap between the scaling of SV services and the completion of PEP within violence response programmes. Innovative implementation science approaches may help to identify and address barriers inhibiting effective PEP delivery and uptake within PVC service delivery programmes. Enhancing PEP uptake and completion can support mitigating the bidirectional relationship between violence and HIV acquisition, particularly among vulnerable populations like adolescents and young adults. Low PEP coverage also reflects missed opportunities, particularly among adolescent girls and young women, who experience disproportionate rates of HIV acquisition.

## 1 INTRODUCTION

Violence is the intentional use of physical force or power, threatened or actual, against oneself or against a group or community that either results in or has a high likelihood of resulting in injury, death, psychological harm, maldevelopment or deprivation [1, 2]. Globally, it is estimated that one in four ever‐married/partnered adolescent girls experiences physical and/or sexual violence (SV) from an intimate partner once in their lifetime [2]. In sub‐Saharan Africa (SSA), the prevalence of experiencing lifetime intimate partner violence (IPV) is 33% [2].

The United States Centers for Disease Control and Prevention (U.S. CDC) defines sexual violence as sexual activity with consent that is not obtained or freely given [1]. Globally, SV has been shown to have a bidirectional positive association with HIV [2−4]. The impact of SV on HIV is seen across the clinical cascade. Across six high‐burden countries in SSA, women exposed to physical and/or sexual IPV in the past 12 months were 3.2 times more likely to have acquired HIV [5]. Several violence against children (boys and girls) have described the associations between SV and HIV, including the ability of survivors of violence to negotiate prevention methods [6−8]. Studies have shown that those who can negotiate prevention methods have a lower risk of HIV acquisition [6−8].

Post‐exposure prophylaxis (PEP) refers to the medications (3‐drug regimen) given to prevent the transmission of HIV following a potential exposure [9, 10]. The World Health Organization (WHO) and the U.S. CDC recommend that persons who experience SV receive PEP within 72 hours (28‐day course) to prevent HIV acquisition [9]. The United States President's Emergency Plan for AIDS Relief (PEPFAR), the most significant bilateral funder of HIV prevention and treatment programmes worldwide (more than 50 countries), makes a substantial investment in violence prevention and response programming [6, 11, 12]. This includes funding for violence prevention and response to more than 20 countries in SSA [12]. PEPFAR‐supported health facilities provide a minimum package of services for SV survivors, which includes care for injuries, rapid HIV testing with referral to care and treatment (as appropriate), PEP, sexually transmitted infection (STI) screening/testing, STI prophylaxis and treatment, emergency contraception and counselling [9, 11, 13].

Despite the expansion and investment in violence service provision in U.S. CDC/PEPFAR‐supported countries, the coverage of PEP use among SV survivors in this context has rarely been explored in low‐resource settings [9, 14−16]. Furthermore, despite WHO guidance for the use of PEP in cases of sexual assault, this remains an understudied field, especially within SSA [9, 14, 17, 18]. PEPFAR's mandated annual reporting on services provides a resource for analysing trends and geographic variations in PEP coverage. This study is the first description of PEP utilization within the U.S. CDC's PEPFAR‐supported violence service delivery programmes in SSA.

## 2 METHODS

PEP data was available from 29 PEPFAR‐supported countries. For this analysis, we restricted the analysis to data from U.S. CDC/PEPFAR‐supported countries in SSA. We analysed PEPFAR's Monitoring, Evaluation, and Reporting (MER) data on post‐violence service provision from October 2018 to September 2023 (fiscal years) across 14 SSA countries. This system semiannually captures aggregate programme data and information on the minimum package of post‐violence services provided in post‐violence clinical settings within PEPFAR‐supported sites. Fifteen SSA countries were excluded from the data due to incomplete data for several years. We included only data reported by U.S. CDC‐supported implementing partners from the 14 countries with data for all or nearly all included years (Cameroon, Cote d'Ivoire, Democratic Republic of Congo, Eswatini, Ethiopia, Kenya, Lesotho, Malawi, Mozambique, Nigeria, Rwanda, Tanzania, Uganda and Zambia). The primary variable of interest was the number of persons receiving post‐violence clinical care categorized by age group, sex and among individuals who experienced SV, completion of PEP (yes/no) [12, 13].

Data also includes the categorization of type of assault: (1) physical and emotional violence (yes/no) and (2) SV (yes/no). To avoid double‐counting, services for an individual who has experienced both sexual and physical and/or emotional violence are only counted under the SV indicator. Those who did not experience SV are counted under the physical and/or emotional violence indicator as appropriate. PEP completion is counted when an individual who has experienced sexual assault receives post‐rape care and a client initiates PEP and self‐reports completing the entire course of treatment according to international guidance and returns for a follow‐up visit [9, 13]. Data on the number of clients initiating and not completing PEP were unavailable and not included in this analysis. All facilities providing the minimum package of services are required to have all providers trained on the provision of post‐violence care (PVC), including PEP. The numbers of clients eligible for PEP and offered but declined PEP are not captured. Additionally, clients may have received PEP and completed the course without reporting back to the clinic. We performed a descriptive analysis using Microsoft Excel.

### 2.1 Ethical review

PEPFAR MER data are covered by a protocol reviewed by the U.S. CDC, deemed not research, and conducted consistent with applicable federal law and U.S. CDC policy (45 C.F.R. part 46.102(l)(2), 21 C.F.R. part 56; 42 U.S.C. Sect. 241(d); 5 U.S.C. Sect. 552a; 44 U.S.C. Sect. 3501 et seq). Only aggregated data with no personal identifying information were used for this analysis; hence there was no need for informed consent by the clients.

## 3 RESULTS

**Figure 1:**
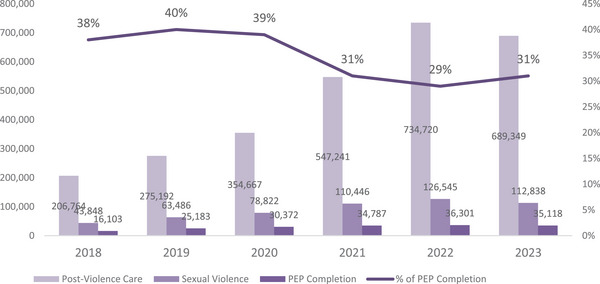
**Annual proportion of PEP completion among clients who received post‐violence care and sexual violence services in 14 countries supported by U.S. CDC/PEPFAR, 2018−2023**. ^*^Sexual violence = denominator for percentage.

From 2018 to 2023, the total client encounters for annually receiving PVC services increased by 233% in the 14 countries, from 206,764 in 2018 to 689,349 in 2023 (Figure 1 and Table S1). Three East African countries, Kenya (*n* = 1,171,259), Tanzania (*n* = 644,574) and Uganda (*n* = 414,031), reported the highest volume of PVC services delivered at U.S. CDC‐supported facilities. During this same period, the total volume of SV services increased by 163%, from 42,848 in 2018 to 112,838 in 2023 (Figure 1, Table 1 and Table S1). Four East African countries, Ethiopia (*n* = 43,363), Kenya (*n* = 101,519), Tanzania (*n* = 100,621) and Uganda (*n* = 137,595), reported the highest volume of SV services delivered. The number of U.S. CDC‐supported facilities providing the minimum service package for PVC increased from 2818 in 2018 to 5220 in 2023 (Table 1). Three countries (Cote d'Ivoire, Lesotho and Mozambique) saw an adverse change in the number of sites providing PVC services. Rwanda was the only country to maintain a constant number of sites providing PVC services. In contrast, the others all had a net increase in the number of facilities providing services (Table 2).

**Table 1:** Number of sites providing the minimum package of post‐violence care service in 14 countries supported by U.S. CDC/PEPFAR, 2018–2023

Country	2018		2019		2020		2021			2022		2023		
	All sites (*n*)	Minimum package (*n*, %)	All sites (*n*)	Minimum package (*n*, %)	All sites (*n*)	Minimum package (*n*, %)	All sites (*n*)	Minimum package (*n*, %)	All sites (*n*)		Minimum package (*n*, %)	All sites (*n*)	Minimum package (*n*, %)	Change (*n*)
Cameroon	344	6 (1.7)	362	10 (2.8)	301	61 (20.3)	144	110 (76.4)	453		101 (22.3)	495	71 (14.3)	65
Cote d'Ivoire	1344	158 (11.8)	1458	107 (7.3)	974	145 (14.9)	1345	138 (10.3)		572	188 (32.9)	588	133 (22.6)	−25
DRC	345	35 (10.1)	357	52 (14.6)	349	50(143)	349	37 (10.6)		380	48 (12.6)	382	39 (10.2)	4
Eswatini	140	30 (21.4)	140	2014.3)	274	16 (5.8)	180	34 (18.9)		174	48 (27.6)	115	35 (30.4)	5
Ethiopia	1131	177 (15.6)	1113	234(21.0)	1184	23519.8)	703	268 (38.1)		1746	328 (18.8)	1859	292 (15.7)	115
Kenya	1457	534 (36.7)	1440	634 (44.0)	1857	713 (38.4)	1416	965 (68.1)		1396	1162 (83.2)	1408	1172 (83.2)	638
Lesotho	111	−	123	−	122	35(28.7)	102	45 (44.1)		99	44 (44.4)	99	32 (32.3)	−3
Malawi	123	−	522	10 (1.9)	538	15(2.8)	582	46 (7.9)		582	57 (9.8)	589	60 (10.2)	50
Mozambique	1006	517 (51.4)	1025	576 (56.2)	1546	43027.8)	1655	415 (25.1)		1703	442 (26.0)	1722	440 (25.6)	−77
Nigeria	1560	306 (19.6)	1742	307 (17.6)	1241	372 (30.0)	1426	426 (29.9)		1421	496 (34.9)	1426	472 (33.1)	166
Rwanda	303	25 (8.3)	299	26 (8.7)	280	25 (8.9)	300	27 (9.0)		271	25 (9.2)	264	25 (9.5)	0
Tanzania	3224	463 (14.4)	3304	613(18.6)	2167	583 (26.9)	2209	862(39.0)		2078	1042 (50.1)	2102	1050 (50.0)	587
Uganda	1650	566 (34.3)	1752	732 (41.8)	1762	764 (43.1)	1860	857 (46.1)		1801	869 (48.3)	2052	870 (42.4)	304
Zambia	1099	1 (0.1)	1128	1 (0.1)	1134	1 (0.1)	1336	152 (11.4)		1212	490 (40.4)	1303	529 (40.6)	528
Total	13,837	2818	14,765	3322	13,729	3445	13,607	4382		13,888	5340	14,404	5220	2402

*Note*: From 2018 to 2023, the number of SV clients completing PEP decreased, with fewer than half completing PEP in 2018 (37%, *n* = 16,103) and in 2023 (31%, *n* = 35,118) (Table 2). More than half of the countries (DRC, Eswatini, Ethiopia, Kenya, Malawi, Rwanda, Tanzania and Uganda) reported that fewer than 50% of SV clients completed PEP over the 6 years.

Abbreviation: DRC, Democratic Republic of the Congo.

**Table 2:** Percentage of all sexual violence clients who completed PEP in 14 countries supported by CDC/PEPFAR, 2018–2023

Country	2018			2019			2020			2021			2022			2023		
	PEP (*n*)	SV (*n*)	Proportion (%)	PEP (*n*)	SV (*n*)	Proportion (%)	PEP (*n*)	SV (*n*)	Proportion (%)	PEP (*n*)	SV (*n*)	Proportion (%)	PEP (*n*)	SV (*n*)	Proportion (%)	PEP (*n*)	SV (*n*)	Proportion (%)
Cameroon	85	86	98.8	45	186	24.2	283	424	66.7	521	817	63.8	462	809	57.1	415	625	66.4
Cote d'Ivoire	351	647	54.3	209	261	80.1	399	497	80.3	540	617	87.5	571	801	71.3	800	872	91.7
DRC	233	463	50.3	222	486	45.7	164	220	74.5	261	432	60.4	508	953	53.3	386	822	47.0
Eswatini	160	677	23.6	104	882	11.8	182	327	55.7	253	481	52.6	365	846	43.1	115	781	14.7
Ethiopia	1398	3311	42.2	2499	5832	42.8	1853	6025	30.8	2509	7511	33.4	3224	10,625	30.3	3258	10,059	32.4
Kenya	3417	7493	45.6	4371	8815	49.6	5541	11,081	50.0	7796	20,655	37.7	6827	29,623	23.0	6402	23,852	26.8
Lesotho	0	0	0.0	0	0	0.0	132	172	76.7	252	420	60.0	304	385	79.0	275	393	70.0
Malawi	0	0	0.0	53	99	53.5	704	1265	55.7	902	1522	59.3	831	1734	47.9	814	1653	49.2
Mozambique	2330	3944	59.1	2779	4500	61.8	2539	4121	61.6	3021	5252	57.5	4458	8087	55.1	5005	8516	58.8
Nigeria	2067	3143	65.8	3745	5742	65.2	5112	6447	79.3	3529	5181	68.1	3081	5920	52.0	1561	2646	59.0
Rwanda	1702	5782	29.4	1411	8284	17.0	2264	7692	29.4	2241	7394	30.3	2547	7227	35.2	2065	6896	29.9
Tanzania	2030	5867	34.6	3907	9040	43.2	5330	18,419	28.9	6100	27,451	22.2	4234	22,488	18.8	4810	17356	27.7
Uganda	1818	9713	18.7	5132	17,721	29.0	4265	18,988	22.5	5233	29,186	17.9	5640	30,982	18.2	5380	31005	17.4
Zambia	512	1722	29.7	706	1638	43.1	1604	3144	51.0	1629	3527	46.2	3249	6065	53.6	3832	7362	52.1
**Total**	16,103	42,848	37.6	25,183	63,486	39.6	30,372	78,822	38.5	34,787	110,446	31.5	36.301	126.545	28.6	35.118	112.838	31

*Note*: Across all years combined, SV clients were young people aged 15–19 (female: 46%, 185,414; male: 41%, 59,618). However, those with the lowest proportion to have completed PEP were 15–19 for males (4%, *n* = 2296) and 40–44 for females (25%, *n* = 2212) (Table 3). Males across all years, except those aged 40–44 (female: 25%; male: 27%), had lower proportions completing PEP than females.

Abbreviations: DRC, Democratic Republic of the Congo; PEP, % post‐exposure prophylaxis completed; PEPFAR, President's Emergency Plan for AIDS Relief; SV, sexual violence.

**Table 3:** Proportion of all sexual violence clients who completed post‐exposure prophylaxis by age band and by sex in 14 countries supported by U.S. CDC/PEPFAR, 2018–2023^a^

Years	Sexual violence		Post‐exposure prophylaxis		Percent of SV clients who completed PEP	
	Female *n* (%)	Male *n* (%)	Female *n* (%)	Male *n* (%)	Female %	Male %
<10	37,123 (9)	15,197 (10)	20,784 (12)	2423 (13)	56	16
10–14	61,030 (15)	22,359 (15)	32,863 (20)	2002 (11)	54	9
15–19	185,414 (46)	59,618 (41)	55,312 (33)	2296 (12)	30	4
20–24	48,666 (12)	18,146 (12)	24,408 (15)	2988 (16)	50	17
25–29	30,276 (7)	11,839 (8)	15,458 (9)	3322 (18)	51	28
30–34	15,554 (4)	7286 (5)	7741 (5)	2226 (12)	50	31
35–39	11,366 (3)	5329 (4)	4593 (3)	1484 (8)	40	28
40–44	8877 (2)	3049 (2)	2212 (1)	826 (4)	25	27
45–49	3308 (1)	1652 (1)	1081 (1)	439 (2)	33	26
50+	4559 (1)	2630 (2)	1948 (1)	618 (3)	43	23

Abbreviations: PEP, post‐exposure prophylaxis; PEPFAR, President's Emergency Plan for AIDS Relief.

^a^ Excludes missing data for sex and age.

## 4 DISCUSSION

Our study demonstrates a significant expansion in PVC service delivery (a 163% increase from 2018 to 2023) in these countries. The total number of sites providing PVC services and the total number of clients reported to have sought services show significant growth during the 6‐year study period. However, our findings also highlight two key areas of concern: the disproportionately low PEP completion rate among women aged 40–44 and poor PEP completion rates among adolescent boys and young men. Over the study period, the percent change in PEP completion decreased by 7 percentage points. This may suggest poor PEP completion within SSA's CDC/PEPFAR‐supported PVC service delivery programmes. However, given the limitations of the data, it is difficult to characterize the change.

We also found that while adolescents make up a large proportion of SV clients, their PEP completion rates are low. This represents a missed opportunity for prevention among a population at high risk for HIV, as women aged 15 years and older account for 61% of all people living with HIV in 2022, with those aged 15–24 years at the highest risk of HIV acquisition [5]. A combination of factors such as quality and availability of services, enabling policies, knowledge of the importance of PVC services, and social attitudes and norms may have played a significant part in service uptake [5].

The findings highlight the poor understanding of PEP initiation and completion among SV clients across the 14 CDC/PEPFAR countries. More than half of the countries in this analysis reported less than 50% PEP completion among those eligible for PEP services. Globally, there are low completion rates of PEP with wide variations, such as in the United States (27.4%), Barcelona (29.0%), Brussels (60.0%) and South Africa (58.5%). However, all indicate suboptimal completion, leaving persons at risk of HIV [16, 19–21]. Barriers to PEP completion are many and include side effects of the drugs, fear of blame for the sexual assault, lack of social support, psychological trauma related to SV and limited knowledge about the importance of completing the entire course of PEP [14, 17, 19]. These findings on PEP completion suggest that more investments in psychological support and adherence counselling for survivors of SV might lead to better outcomes for both adherence and completion [23−25]. One systematic review and meta‐analysis also pointed to higher adherence rates seen in low‐income settings (53.2%, 95% CI 43.5−62.9%) compared with high‐income countries (33.3%, 95% CI 26.0−40.6%) (*p*<0.01) [17]. Such differences have been explained to be due to different attitudes towards HIV and medications, and as such, continued investments in addressing other barriers to PEP completion may lead to higher completion rates [22−24]. Despite fewer Adolescent Girls and Young Women (AGYW) aged 15–24 years acquiring HIV in SSA compared to a decade ago, many still face a substantial risk of acquiring HIV [5, 25], as shown by the large number of young female and male clients seeking SV services in this study. The transmission rate of HIV varies depending on the modality of sexual contact; receptive anal exposure carries the highest risk (0.8−3.0%), followed by receptive vaginal exposure (0.1−0.5%) and oral sex (0.0001−0.01%) [19].

Particularly troubling is the low PEP completion among adolescent boys, highlighting the need for tailored violence prevention and destigmatization efforts. Men and boys often experience severe stigma around experiences of SV in the community and when seeking PVC services. Furthermore, PVC services are usually tailored and designed around the needs of women and heteronormative standards. The findings emphasize the urgent need and demand for PVC services that meet the needs of men and boys.

Low PEP completion is not only a missed opportunity to prevent HIV in the aftermath of SV but also represents a larger opportunity for further intervention and support, including introducing other biomedical options for their longer‐term health needs such as family planning and Pre‐Exposure Prophylaxis (PrEP). Engagement with survivors at the point of PEP completion may present a critical moment to begin to mitigate the cascading effects of violence, given the well‐established link between experiencing violence and HIV risk behaviours.

This study has several strengths: a large‐scale country analysis (14 SSA) with 6‐year trend data, standardized data collections, focus on an understudied area, and programmatic relevance to improve HIV and PVC services.

This study has several limitations. First, we only included data from CDC‐supported facilities through the PEPFAR programme in the selected countries. Thus, we may not represent all persons seeking SV/PVC services in SSA or in PEPFAR programmes supported by other agencies. Second, our data does not account for those clients who experienced sexual assault but were not eligible for PEP initiation (due to late presentation and/or high ongoing risk). This might mean we are underestimating PEP completion by excluding clients not eligible for PEP in the denominator. Third, procedures for recording visits and PEP provision and availability varied across settings. Not all facilities could verify PEP completion by clients, which may have resulted in an underestimation of completion. Fourth, given the high prevalence of HIV in some countries, it is possible that some clients were not eligible for PEP due to their HIV status. Fifth, our data does not allow us to capture the overlap of those clients who may have experienced physical/emotional violence in addition to SV at the same time. Finally, while post‐violence clinical service provision may appear to be increasing, this does not necessarily mean that PEP initiation and completion would increase, as effective violence prevention programmes may simultaneously have been implemented over this period.

## 5 CONCLUSIONS

This study underscores the critical gap between scaling SV clinical services and PEP completion within violence programmes. Enhancing PEP awareness and uptake is essential to implementing successful violence programmes and may help mitigate HIV acquisition, particularly among vulnerable populations like adolescents and young adults. Training of healthcare providers on the eligibility requirements and appropriate prescription of PEP could lead to better rates of PEP completion. Policy and programmatic considerations that may further strengthen programming include strengthening PEP education and awareness campaigns, especially in community settings, and highlighting the importance of timely access to PEP (e.g. Every Hour Matters Campaign), making PEP more readily available including through community‐based platforms and task shifting models, implementing targeted support systems for PEP adherence, especially for AGYW, and improving data collection systems to better track the PEP cascade from eligibility to completion [10, 26]. This study does not explore the barriers to accessing, initiating or completing PEP; however, this could be an area for future analysis. Follow‐up research may help to identify specific barriers to PEP initiation and completion in different demographic groups, develop and test interventions to improve timely access to PEP and adherence, evaluate the effectiveness of integrated violence and HIV prevention services, and investigate the potential of PEP‐to‐PrEP for the population at highest risk of HIV acquisition.

## COMPETING INTERESTS

There are no conflicts of competing interest for authors to declare.

## AUTHORS’ CONTRIBUTIONS

UK and VT encompassed the concept design of the study, data analysis, interpretation of the data and writing the manuscript. All authors contributed to the review and revision of the manuscript. The manuscript underwent a review by all authors, and each one approved the final version.

## FUNDING

This publication has been supported by the United States President's Emergency Plan for AIDS Relief (PEPFAR) through the U.S. Centers for Disease Control and Prevention. The authors received no financial support for the research or authorship of this article.

## DISCLAIMER

The authors declared no potential conflicts of interest regarding the research, authorship and/or publication of this article. The findings and conclusions in this publication are those of the authors and do not necessarily represent the official position of the funding agencies.

## Supporting information




**Table S1**. Post‐violence care service utilization by violence type in 14 countries supported by U.S. CDC/PEPFAR, 2018‐2023.

## Data Availability

Data are available upon request from the authors.
