# Supplementary material for: Poor post‐exposure prophylaxis completion despite improvements in post‐violence service delivery in 14 PEPFAR‐supported sub‐Saharan African countries, 2018–2023
Source: J Int AIDS Soc. 2025 Jun 26;28(Suppl 1):e26469. doi: 10.1002/jia2.26469 (PMC12231646; doi:10.1002/jia2.26469)
Supplement: Supplementary file 1 — Table S1. Post‐violence care service utilization by violence type in 14 countries supported by U.S. CDC/PEPFAR, 2018‐2023. [file JIA2-28-e26469-s001.docx]

Supporting Information

Table S1. Post-violence care service utilization by violence type in 14 countries supported by U.S. CDC/PEPFAR, 2018-2023

| **Country** | **2018** | | | **2019** | | | **2020** | | | **2021** | | | **2022** | | | **2023** | | | **Total** | | | |  |
| --- | --- | --- | --- | --- | --- | --- | --- | --- | --- | --- | --- | --- | --- | --- | --- | --- | --- | --- | --- | --- | --- | --- | --- |
|  | **PVC** | **SV** | **PV** | **PVC** | **SV** | **PV** | **PVC** | **SV** | **PV** | **PVC** | **SV** | **PV** | **PVC** | **SV** | **PV** | **PVC** | **SV** | **PV** | **PVC** | **SV** | **PV** |  |  |
| Cameroon | 205 | 86 | 119 | 305 | 186 | 119 | 692 | 424 | 268 | 1248 | 817 | 431 | 1299 | 809 | 490 | 1017 | 625 | 392 | 4766 | 2947 | 1819 |  |  |
| Cote d'Ivoire | 962 | 647 | 315 | 475 | 261 | 214 | 701 | 497 | 204 | 1026 | 617 | 409 | 2144 | 801 | 1343 | 2200 | 872 | 1328 | 7508 | 3695 | 3813 |  |  |
| DRC | 468 | 463 | 5 | 513 | 486 | 27 | 220 | 220 | - | 432 | 432 | - | 1047 | 953 | 94 | 933 | 822 | 111 | 3613 | 3376 | 237 |  |  |
| Eswatini | 1488 | 677 | 811 | 1329 | 882 | 447 | 548 | 327 | 221 | 733 | 481 | 252 | 1751 | 846 | 905 | 1021 | 781 | 240 | 6870 | 3994 | 2876 |  |  |
| Ethiopia | 3649 | 3311 | 338 | 6297 | 5832 | 465 | 6614 | 6025 | 589 | 8550 | 7511 | 1039 | 12,200 | 10,625 | 1575 | 11,540 | 10,059 | 1481 | 48,850 | 43,363 | 5487 |  |  |
| Kenya | 66,846 | 7493 | 59,353 | 92,943 | 8815 | 84,128 | 117,505 | 11,081 | 106,424 | 218,317 | 20,655 | 197,662 | 360,504 | 29,623 | 330,881 | 315,144 | 23,852 | 291,292 | 1,171,259 | 101,519 | 1,069,740 |  |  |
| Lesotho | - | - | - | - | - | - | 240 | 172 | 68 | 580 | 420 | 160 | 673 | 385 | 288 | 588 | 393 | 195 | 2081 | 1370 | 711 |  |  |
| Malawi | - | - | - | 629 | 99 | 530 | 3346 | 1265 | 2081 | 5045 | 1522 | 3523 | 5762 | 1734 | 4028 | 7393 | 1653 | 5740 | 22,175 | 6273 | 15,902 |  |  |
| Mozambique | 21,526 | 3944 | 17,582 | 23,651 | 4500 | 19,151 | 22,271 | 4121 | 18,150 | 27,542 | 5252 | 22,290 | 40,277 | 8087 | 32,190 | 43,561 | 8516 | 35,045 | 178,828 | 34,420 | 144,408 |  |  |
| Nigeria | 15,865 | 3143 | 12,722 | 29,236 | 5742 | 23,494 | 29,706 | 6447 | 23,259 | 28,123 | 5181 | 22,942 | 32,089 | 5920 | 26,169 | 20,095 | 2646 | 17,449 | 155,114 | 29,079 | 126,035 |  |  |
| Rwanda | 9110 | 5782 | 3328 | 12,739 | 8284 | 4455 | 13,175 | 7692 | 5483 | 13234 | 7394 | 5840 | 13,534 | 7227 | 6307 | 13,163 | 6896 | 6267 | 74,955 | 43,275 | 31,680 |  |  |
| Tanzania | 56,206 | 5867 | 50,339 | 57,004 | 9040 | 47,964 | 93,774 | 18,419 | 75,355 | 138,266 | 27,451 | 110,815 | 147,498 | 22,488 | 125,010 | 151,826 | 17,356 | 13,4470 | 644,574 | 100,621 | 543,953 |  |  |
| Uganda | 28,717 | 9713 | 19,004 | 48,433 | 17,721 | 30,712 | 62,731 | 18,988 | 43,743 | 90,061 | 29,186 | 60,875 | 92,044 | 30,982 | 61,062 | 92,045 | 31,005 | 61,040 | 414,031 | 137,595 | 276,436 |  |  |
| Zambia | 1722 | 1722 | - | 1638 | 1638 | - | 3144 | 3144 | -- | 14,084 | 3527 | 10,557 | 23,898 | 6065 | 17,833 | 28,823 | 7362 | 21,461 | 73,309 | 23,458 | 49,851 |  |  |
| **Total** | 206,764 | 42,848 | 163,916 | 275,192 | 63,486 | 211,706 | 354,667 | 78,822 | 275,845 | 547,241 | 110,446 | 436,795 | 734,720 | 126,545 | 608,175 | 689,349 | 112,838 | 576,511 | 2,807,933 | 534,985 | 2,272,948 |  |  |
| Abbreviations: DRC = Democratic Republic of the Congo, PVC= Post-Violence Care, SV = Sexual Violence, PV = Physical Violence, PEPFAR=President’s Emergency Plan for AIDS Relief | | | | | | | | | | | | | | | | | | | | | | | |
